# Environment Responsive Metal–Organic Frameworks as Drug Delivery System for Tumor Therapy

**DOI:** 10.1186/s11671-021-03597-w

**Published:** 2021-09-03

**Authors:** Chao Yan, Yue Jin, Chuanxiang Zhao

**Affiliations:** 1 The Affiliated Huai’an Hospital of Xuzhou Medical University and The Second People’s Hospital of Huai’an, No. 62, Huaihai Road (S.), Huai’an, 223002 China; 2 School of Medical Technology, Jiangsu College of Nursing, Huai’an City, Jiangsu Province China

**Keywords:** Nanoparticles, Metal–organic frameworks, Unique tumor microenvironment

## Abstract

Nanoparticles as drug delivery systems can alter the drugs' hydrophilicity to affect drug uptake and efflux in tissues. They prevent drugs from non-specifically binding with bio-macromolecules and enhance drug accumulation at the lesion sites, improving therapy effects and reducing unnecessary side effects. Metal–organic frameworks (MOFs), the typical nanoparticles, a class of crystalline porous materials via self-assembled organic linkers and metal ions, exhibit excellent biodegradability, pore shape and sizes, and finely tunable chemical composition. MOFs have a rigid molecular structure, and tunable pore size can improve the encapsulation drug's stability under harsh conditions. Besides, the surface of MOFs can be modified with small-molecule ligands and biomolecule, and binding with the biomarkers which is overexpressed on the surface of cancer cells. MOFs formulations for therapeutic have been developed to effectively respond to the unique tumor microenvironment (TEM), such as high H_2_O_2_ levels, hypoxia, and high concentration glutathione (GSH). Thus, MOFs as a drug delivery system should avoid drugs leaking during blood circulation and releasing at the lesion sites via a controlling manner. In this article, we will summary environment responsive MOFs as drug delivery systems for tumor therapy under different stimuli.

## Introduction

Tumor is a multifactorial disease with high mortality and recurrence rates that threaten human health [1]. In clinics, chemotherapeutic drugs and surgery applied for tumor therapy have achieved tumor inhibition but often with serious side effects, which promoted us to develop superior therapeutic methods [2, 3]. Over the past decades, nanocarriers have been developed for tumor imaging, theranostics and therapy [4].

In all kinds of nanocarriers, metal–organic frameworks (MOFs) have attracted increasing attention, as they can be stimulated by different environment [5, 6]. MOFs, as a class of high crystalline inorganic–organic porous materials, consist of metal ions or clusters linked by organic bridging ligands and have attracted tremendous attention in recent years in different fields [7]. Earlier than the 1990s, MOFs has been widely applied in gas storage, separation catalysis, energy conversion, luminescence and chemical sensing, and biomedical field, due to their finely tunable chemical composition, pore shape and size, morphology, large surface area and excellent biodegradability [8, 9].

MOFs have organic active sites and accessible, opening porous architectures, chemical stability, and sufficient thermal effects [10]. Thus various functional groups can integrate into MOFs via three strategies: encapsulation, grafting, and infiltration, which can improve their biocompatibility, solubility and interactivity with a target molecules [11]. In particular, the encapsulation approach through coprecipitation and biomimetic mineralization method is the rapid and convenient approach using the organic ligands and metal ions to achieve one-step embedding of drugs into MOFs [12, 13]. Inspired from these excellent merits, various methods have been made to identify its feasibility and effectiveness of utilize. However, MOFs can easily grow at different substrates to form multifunctional complexes [14].Thus, some therapeutic agents can directly incorporate into MOFs via synthesis progress, which can circumvent crystal growth problems when applying pre-functionalized ligands [15, 16]. Such a strategy provides a high atomic economy and leads to extremely satisfactory drug payloads [14].

Although MOFs as drugs delivery system for tumor therapy has unparalleled advantages, their application has been restricted by many intractable drawbacks. For example, MOFs are a complicated synthetic progress, eliminated by the body’s immune system, and has a short half-life in the blood [17–19]. In this article, we will summarize some basic environment stimuli-responsive MOFs to enhance tumor therapy and review the current state of the tumor theranostics.

### pH/ATP Responsive

Zeolitic imidazolate frameworks (ZIFs), as the specific subclass of MOFs, have tunable pore size, ultra-large surface area, and facile synthesis progress. ZIFs are synthesized via biomimetic mineralization and coprecipitation used as the ideal drug carrier for tumor theranostics [20]. Moreover, ZIFs nanoparticles can achieve endosome escape, ascribed to the protonation of the imidazole-2-carboxaldehyde (2-ICA) in the acidic endosome that drives the "proton sponge" effect [21].

**Fig. 1:**
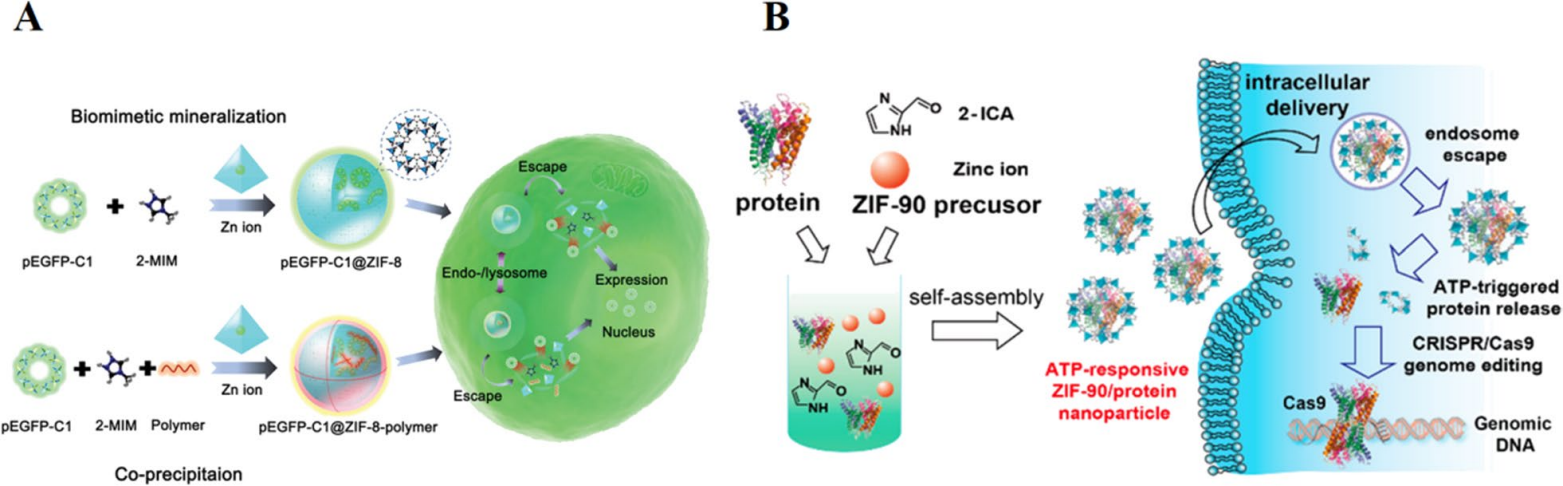
**A** Schematic representation for synthesis of pEGFP-C1@ZIF-8 nanostructures and pEGFP-C1@ZIF-8-polymer nanostructures via biomimetic mineralization and coprecipitation method, respectively, and their cellular delivery and expression process [26]. Copyright 2019 American Chemical Society. **B** Schematic illustration of the self-assembly of ZIF-90/protein nanoparticle and ATP-triggered protein release from ZIF-90 nanoparticle inside cells [29]. Copyright 2019 American Chemical Society

Gene therapy has attracted great attention both in basic and clinical research for tumor therapy in the past decades [22]. However, naked nucleic acids are easily degraded by the blood serum nuclease. They are too large and fragile to pass through cell membrane resulting in unsatisfactory therapy outcomes [23, 24]. Zeolitic organic framework-8 (ZIF-8) is fabrication via the one-pot method by low toxicity metal ions (Zn^2+^) and 2-methylimidazole (2-Mim) under mild conditions. It has excellent encapsulation capability and protects genes against enzyme degradation [25]. Li and his co-workers provided a one-step approach to load large plasmid DNA (pDNA) molecules into ZIF-8 and ZIF-8 polymer systems through biomimetic mineralization and coprecipitation approach (Fig. 1A shown) [26]. ZIF-8 and ZIF-8 polymer systems exhibit excellent encapsulate capability, well distribution of loading pDNA against the enzymatic degradation, and better pH-responsive release. Importantly, higher molecule weight (MW) cationic polymer (PEI) functionalization MOFs-polymer system enhances the electrostatic interaction with pDNA, improving cellular uptake and endo-/lysosomal escape resulting in remarkable gene expression [27]. Thus, these ZIF-8 and ZIF-8 polymer-based nanocarriers for gene therapy offer an economical, convenient and rapid approach to encapsulate gene molecules for effective intracellular transportation and expression.

The concentration of ATP is lower than 0.4 mM in the extracellular. However, the concentration is upregulated in the cytosol or diseased cells (1–10 mM) [28]. Thus, the ATP-responsive drug delivery system will open a new window for advanced drug delivery for targeting disease therapy. Figure 1B shown, Yang et al. reported ATP-responsive zeolitic imidazole framework-90 (ZIF-90) as an ideal nanocarrier for cytosolic protein delivery, which was simply prepared via mixing Zn^2+^ and imidazole-2-carboxaldehyde (2-ICA) at the protein solution [29]. At the tumor sites, as-prepared ZIF-90/protein MOFs will gradually degrade to release preload protein due to the competitive coordination between the Zn^2+^ and ATP that disassembles ZIF-90 and the releasing protein can effectively inhibit cancer cells growth. Thus, we can speculate that ZIP-90 MOFs can encapsulate molecular weighted protein regardless of molecular weight and protein size. This includes superoxide dismutase and bovine serum albumin with minimal effects on protein function for tumor therapy.

Due to the abnormal TME, this ATP-responsive protein delivery system illustrated in this section not only expands the chemistry of MOFs in biomedical applications, but also opens up a new window for protein delivery and genome editing techique for targeting disease therapy.

### Light Responsive

As a "green" approach, photothermal therapy has minimal toxicity to surrounding tissues, widely applied in tumor therapy [30, 31]. High temperatures can induce severe irreversible damage to tissues when the temperature sustains over 44 °C. It is enough to cause cell membrane damage, mitochondrial dysfunction, and disruption RNA synthesis to induce cell death [32]. Unlike normal tissues that can dissipate heat and keep the temperature constant by blood circulation via neuromodulation, locking of autonomous regulatory function made tumor tissues a heat reservoir. This provides a huge advantage for subsequence photothermal therapy [33].

**Fig. 2:**
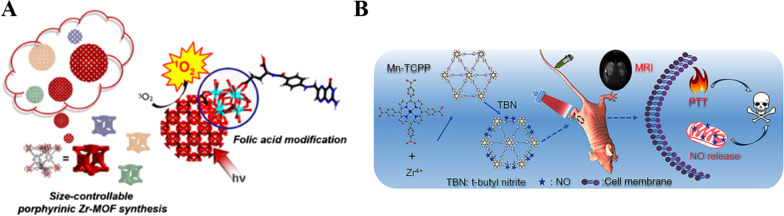
**A** Illustration of PCN-224 structure. 6-connected Zr_6_ cluster (Zr_6_O_4_(OH)_4_(H_2_O)_6_(OH)_6_(COO)_6_), tetratopic linker (tetrakis (4-carboxyphenyl)porphyrin (H_2_TCPP)), and 3D nanoporous framework of PCN-224. (b) A cubic unit of PCN-224 and schematic illustration of spherical PCN-224 nanoparticles on the basis of construction of cubic units, yielding different sizes [39]. Copyright 2018 American Chemical Society. **B** Scheme for the synthesis of the NMOF–SNO nanocomposite and the NIR light-triggered NO release and PTT [42]. Copyright 2018 American Chemical Society

Based on these merits mentioned above and poor heat-dissipating ability, photo-based therapy may be suitable for tumor therapy. Photodynamic therapy (PDT) is the typical approach of photothermal therapy, which is constituted by three basic elements (near-infrared light irradiation, plenty of oxygen, and photosensitizers) [34]. Near-infrared light irradiation (NIR light) as external stimulus exhibits high spatial and temporal control of local heating with minimal adverse side effects [35, 36]. PSs utilized surrounding oxygen to generate poisonous reactive oxygen species (ROS) to destroy cancer cells under laser irradiation [37, 38]. As shown in Fig. 2A, Park et al. designed Zr(IV)-based porphyrinic metal–organic framework (Zr-MOF) that can generate ROS under NIR light [39]. Up injection into the body, Zr-MOF can accumulate at the tumor tissues via the enhanced permeability and retention (EPR) effects. However, the targeting ability was not satisfactory, which could increase unnecessary side effects [40]. Thus, Zr-MOF was further modified with folic acid, improving Zr-MOF targeting ability during blood circulation time and enhancing PDT efficacy.

With the assistance of contrast agents, this can provide precise therapy navigation and determine the suitable therapeutic time [41. As shown in Fig. 2B, Zhang and his co-workers developed Mn-porphyrin MOFs via self-assembling of Mn-tetrakis (4- carboxyphenyl) porphyrin and Zr^4+^ ions, which endow Mn-porphyrin MOFs with the magnetic resonance imaging (MRI) and photothermal conversion capacity without increasing tedious synthesis progress [42]. These novel MOFs can further conjugate with the type heat unstable NO donor s-nitrosothiol (SNO) [43]. Therefore, this MOFs platform can achieve the photothermal and MRI-guided NO synergistic treatment. MOFs-SNO can efficiently accumulate at the tumor areas through intravenous injection, and realize high photothermal conversion ability for PTT and control NO release for NO synergistic therapy with less photo-damage. Thus, theranostic agents integrated into the MOFs are a feasible approach for enhancing the diagnosis and provide precise therapy navigation and determine the suitable therapeutic time.

Due to free porphyrin has optical properties, when porphyrin integrated into the MOFs, the obtained porphyrin MOFs has fluorescence imaging and PDT, which will opens new opportunities for next-generation tumor theranostics.

### H_2_O_2_ Responsive

**Fig. 3:**
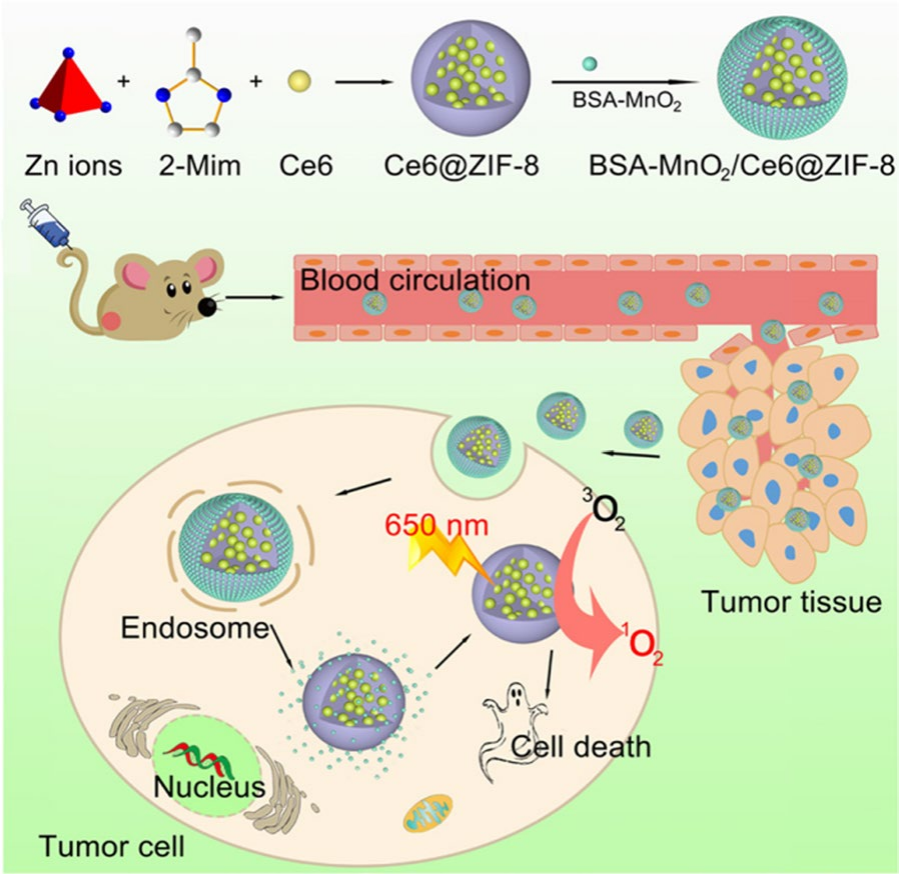
Schematic illustration for the Formation of a BSA-MnO_2_/Ce6@ZIF-8 Nanoplatform and Schematic Illustration Showing the TME Responsiveness and Generation of ROS Irradiation upon 650 nm NIR Laser for MRI-Guided Photodynamic Cancer Treatment [53]. Copyright 2019 American Chemical Society

High levels of H_2_O_2_, hypoxia, low pH value, and high concentration glutathione (GSH) are common feature in the tumor microenvironment (TME) [44–46]. Therefore, ameliorating or changing unique TME can inhibit tumor growth and enhance therapeutic effects [47, 48]. Many literatures have reported that MnO_2_ has nanoenzyme activity can decompose into Mn^2+^ and release amount O_2_ under the circumstances of H_2_O_2_, which can increase oxygen concentration inside the solid tumors and generation abound reactive oxygen species (ROS) under laser irradiation [49, 50]. ROS, as the intracellular chemical substrate, can modulate cell signal and play an important role in the cell cycle [51]. Important, cancer cells are more sensitive to high levels of ROS and susceptible to apoptosis [52]. As Fig. 3 shows, Sun et al. constructed bovine serum albumin-MnO_2_/chlorin e6@ZIF-8 (BSA-MnO_2_/Ce6@ZIF-8) nanosystem exhibits pH/H_2_O_2_ controllability for O_2_ production capacity, which offered a safe and efficient PDT therapy administration progress [53]. Photosensitizer chlorin e6 (Ce6) loading into the ZIF-8 can resolve the low dissolubility problem in the aqueous environment and generate ROS to induce cancer cells apoptotic and necrotic under 650 nm laser irradiation. Bovine serum albumin (BSA)-MnO_2_ decorated into the surface of Ce6@ZIF-8, the obtained BSA-MnO_2_/Ce6@ZIF-8 has excellent dispersibility, low toxicity, sufficient oxygen generation ability, and minimal side effects in vitro/in vivo. This well-prepared BSA-MnO_2_/Ce6@ZIF-8 nanosystem possesses a pH/H_2_O_2_-sensitive capacity and follows the MRI-guided PDT, which holds enormous potential for more accurate diagnosis and improvements to the antitumor effects.

### GSH Responsive

PDT has achieved a distinct advantage in tumor therapy; a high concentration of glutathione (GSH) in cancer cells (2–10 mM) not only resists PDT, radiotherapy, and chemotherapy, but also serves as an antioxidant to scavenge cellular ROS and severely compromises the PDT application [54, 55]. More specifically, it has been reported that excessive ROS can cause inflammation to tumor tissues and serious phototoxicity to normal tissues [56, 57]. Thus, it is urgent to develop an intelligent MOFs system, which can simultaneously achieve PSs-mediated ROS generation and reduce the negative effects of intracellular GSH on the cytotoxicity of ROS at the tumor areas.

**Fig. 4:**
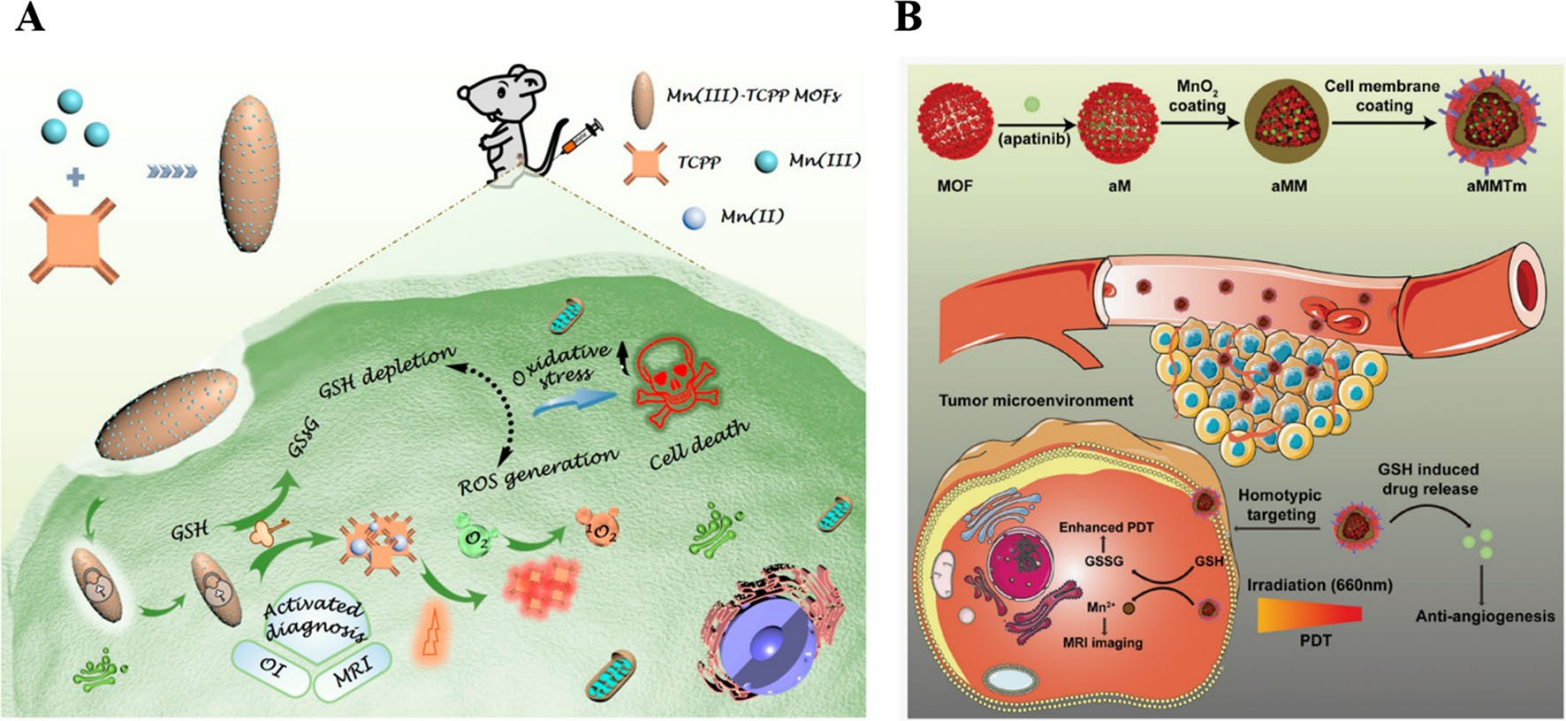
Schematic illustration of an endocytosis Mn(III)-sealed MOF nanosystem for MRI and OI-guided PDT by controlled ROS generation and GSH depletion after being unlocked by overexpressed GSH in tumor cells [58]. Copyright 2019 American Chemical Society. **B** Schematic illustration of aMMTm preparation and proposed combination therapy of PDT and antiangiogenesis[55]. Copyright 2019 WILEY–VCH Verlag GmbH & Co. KGaA, Weinheim

In order to meet these requirements, Wan et al. provided a GSH-unlocked Mn (III)-sealed MOFs nanosystem to undergo a reductive disintegration by high-level GSH in tumor sites. This can control GSH depletion and ROS generation exhibited comprehensive tumor inhibition by improving the therapeutic effects of PDT (Fig. 4A shown) [58]. However, the major challenge of MOFs in medical applications are their unfavorable biocompatibility and short blood half-life. Thus many strategies to optimize MOFs in vivo application have attracted significant attention [59]. Inspiring from circulating blood cells, biomimetic cloaking with the plasma membrane is a powerful approach to coordinate the fate of inorganic nanomaterials in vivo [60–62]. As shown in Fig. 4B, Min and his co-colleagues illustrated multifunctional biomimetic MOFs nanoparticles with 4T1 breast cancer cell membrane camouflage for synergic anticancer therapy of PDT and antiangiogenesis [55]. Such design can keep the surface proteins inherited from the donor cells and endow 4T1 cells decorated MnO_2_ coated porphyrinic Zr-MOF loaded vascular endothelial growth factor receptor 2 MOFs (aMMTm) additional biological function to escape macrophage recognition and target tumor tissue via homotypic affinity in vivo. More importantly, MnO_2_ decorated into the surface of MOFs to neutralize high intratumoral levels of GSH and H_2_O_2_ to ameliorate the unique tumor microenvironment, which can boost the PDT outcomes. When the MnO_2_ shell was gradually degraded, the released Mn^2+^ can act as an MRI contrast agent and apatinib neutralized the PDT-induced revascularization and prevented tumor progress. We believe that this multifunctional drug delivery system has enormous potential capacity in mechanism-based customization of antitumor therapy.

The as-fabricated biomimetic nanosystem for dual imaging-guided synergistic tumor therapy was a simple theranostic system, which would pave a new avenue for tumor diagnosis and therapy.

### Hydrogen Sulfide (H_2_S) Responsive

**Fig. 5:**
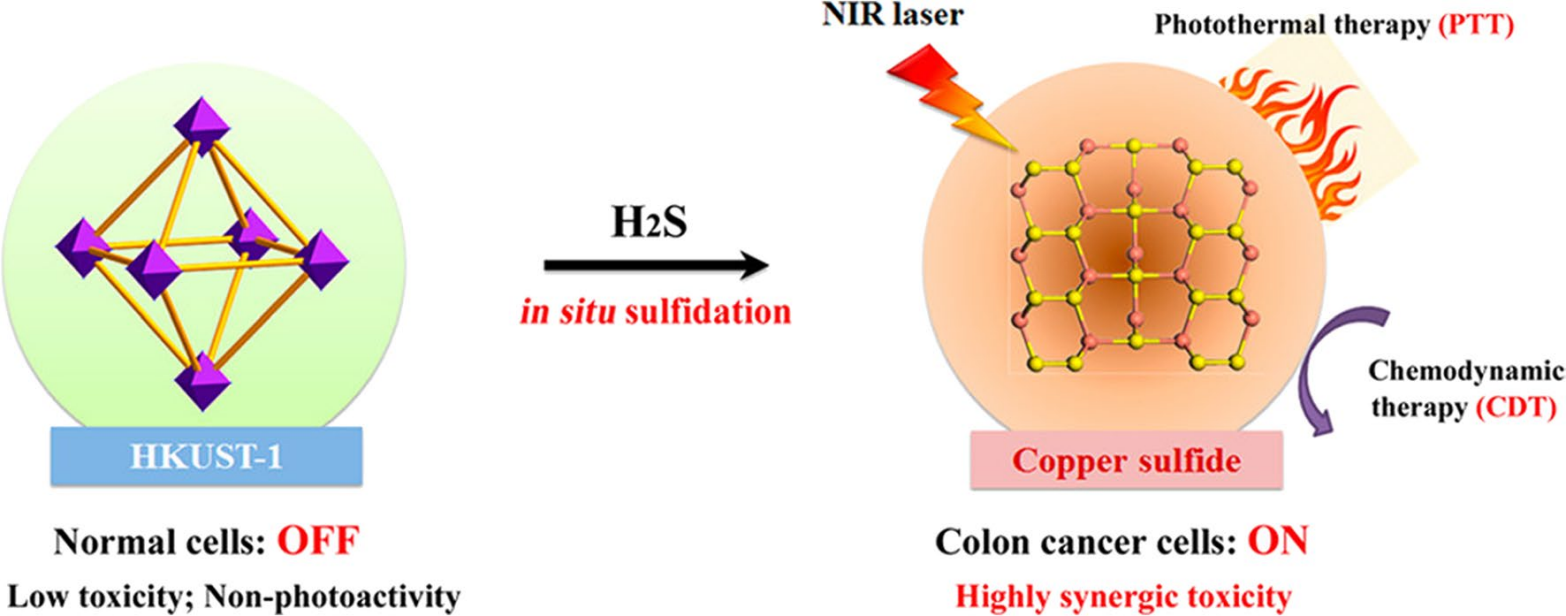
Schematic Illustration of the H_2_S-Triggered Transformation of Non-photoactive Cu-MOFs Nanoenzyme into an Near-NIR-Activatable Photothermal Agent by in Situ Sulfifidation Reaction and Its Further Synergic Photothermal and Chemodynamic Therapy for Colon Cancer [68]. Copyright 2020 American Chemical Society

Endogenous hydrogen sulfide (H_2_S), as the third gasotransmitter, is generated from the enzyme system of cystathionine β-synthase via the catalysis process [63, 64]. Cu-based MOFs have a strong binding ability of Cu^2+^ with S^2−^, and their inherent activity of Cu^2+^ possessed higher catalytic activity in acid [65]. In recent years, Cu-MOFs have been exploited to detect the toxic H_2_S gas in the serum or solution [66]. Thus, H_2_S can be recognized as a specific "target signal" for ovarian and colon tumor diagnosis and therapy [67]. As shown in Fig. 5, Li and his co-workers provided endogenous H_2_S-activated Cu-MOF is in the "OFF" state and no obvious adsorption at the NIR region. However, when Cu-MOFs entered into the colon tumor tissues where H_2_S was overexpressed, Cu-MOFs can change into the “ON” state by reacting with high levels of H_2_S concentration to generate photoactive copper sulfide with stronger NIR absorption, which promoted photothermal therapy (PTT) [68]. Cu-MOFs has the mimicking-peroxidase activity and reacted with overexpressed H_2_O_2_ to produce toxical hydroxyl radical for hemodynamic therapy after endocytosed by the cancer cells [69]. Thus, H_2_S-triggering ‘turn-on’ strategy exhibits excellent antitumor outcomes and avoid unnecessary side effects in tumor therapy. This H_2_S-triggered nanocarrier can significant inhibit colon cancer cells grown in vivo, and this biomarker triggered therapeutic agents show enormous potential for tumor diagnosis and therapeutic.

### Perspectives

MOFs as drug delivery systems for tumor therapy, show unparalleled advantages due to their intrinsic features, including structural tenability, high porosity, multifunctionality, and biocompatibility. Although MOFs have achieved impressive progress in the biomedical field, several key problems need to be addressed before MOFs can be permitted to clinical translation stages. These include complexed synthesis, early clearance by body immune system, system toxicity, unsatisfactory pharmacokinetics and biodistribution, off-target accumulation, and untimely drug release ability.

In order to solve these multileveled problems, biomimetic cloaking with the plasma membrane is a powerful strategy to tune the fate of MOFs in vivo. All kinds of cell membranes have been widely applied to camouflage MOFs. This biomimetic approach can make up MOFs with the biointerface of cell membranes, which can keep the surface proteins inherited from the donor cell, reduce their elimination from the body immune system to prolong their half-life in the blood, and enhance MOFs accumulated at the tumor tissues via permeability and retention effects. Based on these merits, cell membrane and MOFs combined biomimetic platforms to maximize the therapeutic agents to tumor tissues and effectively achieve tumor therapy.

Especially, the distorted cancer blood vessels and cancer cells' rapid proliferation would cause low oxygen concentration and acidification in the tumor microenvironment (TME). Hypoxia, low pH, and high GSH concentration are the common features in the TEM, which promote cancer metastasis and angiogenesis and lead to therapeutic resistance and compromise therapy outcomes. Developing environment responsive and intelligent MOFs triggering by tumor microenvironment is a feasible approach for the substantial elevation in precise diagnosis, and reduction in unnecessary side effects in tumor therapy.

## Conclusion

In this article, we summarized various kinds of MOFs based on their unique mechanisms and structures. Complex design, high operating costs, and lengthy preparation steps, are obstacles MOFs encounter in real application to the clinical field. Ultimately, targeting delivery, low to none toxicity, and outstanding therapeutic effects are the critical factors for successful translating MOFs to clinical application.

## Data Availability

Not applicable.
